# ﻿*Synotislhozhagensis* (Asteraceae, Senecioneae), a new species from south-eastern Xizang, China, based on morphological and molecular evidence

**DOI:** 10.3897/phytokeys.259.156141

**Published:** 2025-07-01

**Authors:** Guo-Xing Peng, Liao-Cheng Zhao, Ming Tang, Jun Deng, Hao-Ming Zhan, Zhuo-Ni Zhang, Qiang Wang

**Affiliations:** 1 College of Forestry, Jiangxi Agricultural University, Nanchang 330045, Jiangxi, China; 2 Laboratory of Systematic Evolution and Biogeography of Woody Plants, College of Ecology and Nature Conservation, Beijing Forestry University, Beijing, 10083, China; 3 Jiangxi Provincial Key Laboratory of Improved Variety Breeding and Efficient Utilization of Native Tree Species, Jiangxi Agricultural University, Nanchang 330045, Jiangxi, China; 4 Jiangxi Provincial Key Laboratory of Conservation Biology, Jiangxi Agricultural University, Nanchang 330045, Jiangxi, China; 5 State Key Laboratory of Plant Diversity and Specialty Crops, Institute of Botany, Chinese Academy of Sciences, Beijing 100093, China; 6 National Botanical Garden, Institute of Botany, Chinese Academy of Sciences, Beijing 100093, China; 7 College of Life Sciences, University of Chinese Academy of Sciences, Beijing 100049, China

**Keywords:** Compositae (Asteraceae), morphology comparison, phylogenetic analysis, taxonomy, Xizang (Tibet)

## Abstract

*Synotislhozhagensis*, a new species belonging to Senecioneae (Asteraceae), is described and illustrated herein after being discovered during biodiversity surveys in Lhozhag County, Xizang Autonomous Region, China. This species is similar to *S.acuminata* (Wall. ex DC.) C.Jeffrey & Y.L.Chen, but differs mainly in having the ray floret absent (vs. 1), the leaf margins irregularly coarsely serrate (vs. finely mucronulate-serrate) and the phyllaries five (vs. three or four). It is also allied to *S.glomerata* C.Jeffrey & Y.L.Chen in growth habit, leaf blade morphology and inflorescence structure, but is distinguished by the corymbs loose and spreading (vs. dense and glomeruliform), the ligulate floret absent (vs. one or two) and the disc florets five (vs. three or four). Phylogenetic analysis, based on ITS sequences, confirms its distinct taxonomic status within *Synotis*. A line drawing and colour plates, a geographic distribution map, a detailed comparison table including the aforementioned three species and the provisional IUCN status are provided.

## ﻿Introduction

*Synotis* (C.B. Clarke) C. Jeffrey & Y.L. Chen (Asteraceae, Senecioneae), a genus endemic to the Central Himalayas (Jeffrey and Chen 1984; Nordenstam 2007; Tang et al. 2013a, b; Tang 2014; Tong et al. 2017; Zhao et al. 2023; Jin et al. 2024; Liu et al. 2024), comprising approximately 62 species primarily distributed in the Sino-Himalayayan Region. Southwest China serves as its centre of diversity, hosting over 50 documented species (Chen et al. 2011; Tang 2014; Liu et al. 2021; Fan et al. 2022; Liu et al. 2024). Despite the foundational taxonomic framework established by Jeffrey and Chen (1984) through comprehensive revisions, species delimitation within this genus remains challenging due to the high uniformity of morphological traits (Tong et al. 2017; Liu et al. 2024). Tang (2014) conducted an integrative taxonomic study combining morphological analyses (including ultrastructural observations via scanning electron microscopy, SEM), cytological data and molecular phylogenetics on the genus and refined the subordinate taxonomic system, pointed out the five series belonging to two sections, i.e. sect. Synotis and sect. Atractylidifoliae C. Jeffrey & Y. L. Chen, were included in the genus. Li et al. (2018) transferred *Seneciokarelinioides* C. Winkl. to *Synotis* based on evidence from morphology, karyology and ITS/ETS sequence data and recombined *Sen.karelinioides* as Syn.sect.Karelinioidei (O. Fedtsch. & B. Fedtsch. ex Schischk.) C. Ren, Lazkov & I. D. Illar., treating Synotissect.Atractylidifoliae as a synonym of the new combination.

During biodiversity surveys conducted in Lhozhag County, Xizang Autonomous Region in August 2024, we discovered a morphologically distinct population of *Synotis* near Lakang Town. Initial morphological comparisons showed that its leaf characteristics were congruent with *S.acuminata*, whereas its floral traits (smaller capitula with five phyllaires) were more aligned with *S.glomerata*. Subsequent targeted morphological examinations, along with molecular phylogenetic analyses using ITS sequence data, confirmed the phylogenetic distinctiveness of the taxon within the genus. We hereby formally describe this previously undocumented species as *Synotislhozhagensis* sp. nov., with its systematic placement resolved through integrative taxonomic assessment.

## ﻿Material and methods

### ﻿Morphological analyses

Field observations of the natural habitat and habit of the new taxon were systematically recorded. Comparative studies of the new species and its putative allies were conducted by field observation and examination of specimens, focusing on diagnostic characters: leaf and inflorescence shape, capitula size, number of phyllaries and presence or absence of ray florets. Key morphological traits of five specimens were documented using a standardised digital imaging system (Olympus TG-6) according to the protocols of Zhao et al. (2023).

### ﻿Species sampling, DNA extraction and sequencing

Based on previous phylogenetic studies in Senecioneae (Pelser et al. 2007, 2010; Zhao et al. 2023; Jin et al. 2024), we selected 43 samples encompassing 41 species and one subspecies, across seven genera, including 14 *Synotis* and 23 *Senecio* taxa, with *Abrotanellaemarginata* Cass. (subtribe Abrotanellinae) chosen as the outgroup.

Furthermore, we generated new sequences for the newly-discovered species (vouchers.01/02) using the modified CTAB method (Doyle and Doyle 1987). Low-coverage genomic data (> 2 Gb per sample) were obtained through Illumina NovaSeq PE150 sequencing (Novogene, Beijing), employing library preparation following a 150 bp paired-end strategy. Raw sequencing data were processed using Trimmomatic v. 0.39 (Bolger et al. 2014) to remove unpaired and low-depth reads, enhancing the accuracy and quality of the assembly. Filtered data were assembled into nuclear ribosomal DNA (nrDNA) sequences using GetOrganelle v. 1.7.7(Jin et al. 2020) and ITS sequence was extracted from nrDNA sequence with ITSx (Bengtsson et al. 2013).

In addition to sequencing the newly-described species, nuclear ribosomal (ITS) regions of its putative relatives, *S.glomerata* and *S.acuminata*, were amplified using the ITS4 and ITS5 primer pairs (White et al. 1990) following the protocols of Tang (2014). ITS sequences of the remaining species were retrieved from the GenBank database. Voucher information and GenBank accession numbers for the material used in this study are provided in Appendix 1 (Table A1).

### ﻿Phylogenetic analyses

Data processing and analysis were performed using PhyloSuite v.1.2.3 (Zhang et al. 2020). Multiple sequence alignment was carried out with MAFFT v.7 (Katoh and Standley 2013). Maximum Likelihood (ML) inference was conducted in IQ-TREE v.2.2.0 (Nguyen et al. 2015) under the SYM+G4 model, selected by ModelFinder (Kalyaanamoorthy et al. 2017), based on the corrected Bayesian Information Criterion. Node support was derived from 1,000,000 ultrafast bootstrap replicates (Minh et al. 2013). Bayesian Inference (BI) was executed using MrBayes v.3.2.7 (Ronquist et al. 2012) under the SYM+G4 model, with two independent runs of 2,000,000 generations each, sampling every 1,000 generations. Convergence was assessed by monitoring the average standard deviation of split frequencies (< 0.01) and ensuring effective sample sizes (ESS) exceeded 200 for all parameters. After discarding the initial 25% of samples as burn-in, posterior probabilities (PP) were calculated from the remaining trees. Bootstrap percentage (MLBS) values ≥ 70 (Huelsenbeck et al. 1993) and PP values ≥ 0.90 (Leaché et al. 2002) were considered strong support. Finally, the phylogenetic trees were visualised using FigTree v.1.4.

## ﻿Results and discussion

### ﻿General morphology

**Figure 1.**
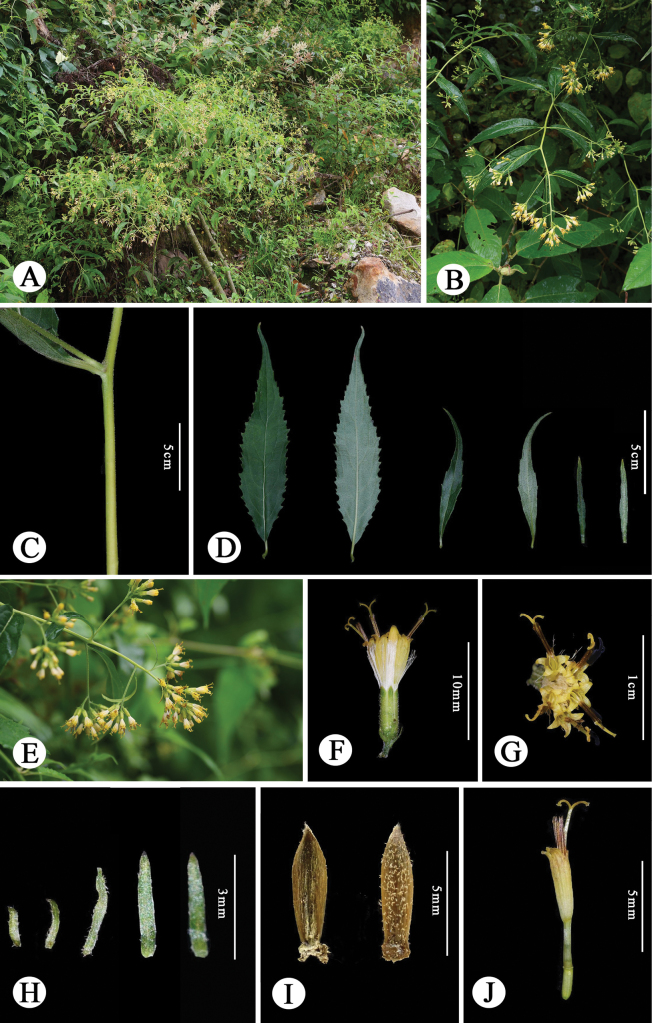
*Synotislhozhagensis* in its natural habitat in Shannan, Xizang, China. **A.** Habitat; **B.** Habit; **C.** Portion of stem (sparsely tomentose); **D.** Leaf blades (left one and two: adaxial surface and abaxial surface; left three and left four: adaxial surface and abaxial surface; left five and left six: adaxial surface and abaxial surface); **E.** Synflorescence; **F.** Capitulum, lateral view; **G.** Capitulum, apical view; **H.** Outer phyllaries (= bracts of calyculus); **I.** Phyllary, adaxial (left) and (right) surfaces; **J.** Disc floret. Photographed by H.L. Zheng & X.T. Ma.

*Synotislhozhagensis* closely resembles *S.glomerata* and *S.acuminata* morphologically, but is distinguished by a suite of macroscopic morphological traits: *S.lhozhagensis* is relatively taller (100–250 cm vs. 40–150 cm in *S.acuminata* and ≤ 120 cm in *S.glomerata*), leaves with irregularly deep obtuse-acuminate serrate margins (vs. remotely mucronate-serrulate margins in *S.acuminata* and *S.glomerata*), capitula lacks ray florets (vs. one in *S.acuminata* and one or two in *S.glomerata*), disc florets five (vs. two or three in *S.acuminata* and three or four in *S.glomerata*) (Figs 1, 6, 7, Table 1). These differences highlight *S.lhozhagensis* as a unique taxon within the genus.

**Table 1.** Morphological comparison of *Synotislhozhagensis* sp. nov., *S.glomerata* and *S.acuminata*.

Character	*Synotislhozhagensis*	*S.glomerata*	*S.acuminata*
**Stem**	100–250 cm tall, white-tomentose when young	≤ 120 cm tall, yellowish-brown tomentose	40–150 cm tall, yellowish-brown pubescent
**Leaf size**	6–13 × 1.5–3 cm	6–22 × 1.5–6 cm	8–18 × 1.5–3.5 cm
**Leaf margin**	Irregularly serrate-dentate	Finely serrulate with mucronate teeth	Sparsely serrulate
**Lateral veins**	4–6 pairs	8 or 9 pairs	5 or 6 pairs
**Inflorescence type**	Loose thyrsoid-paniculate	Dense globose corymb (20–25 capitula)	Compound corymb
**Phyllaries**	5, oblong, silvery-puberulent	5, oblong, apex obtuse	3 or 4, linear-oblong, glabrous
**Ray florets**	Absent	1 or 2, corolla liliform, 3–5 mm long	1, corolla linear, 5–7 mm long
**Disc florets**	5, pale yellow, corolla 8 mm long	3 or 4, yellow, corolla 7 mm long	2 or 3, yellow, corolla 7 mm long

### ﻿Phylogenetic analyses

Both Maximum Likelihood (ML) and Bayesian Inference (BI) phylogenetic analyses yield congruent topologies, with the ML tree topology being presented here (Fig. 4). The genus *Synotis* formed a strongly supported monophyletic clade (MLBS = 99, PP = 1.00), consistent with previous phylogenetic studies (Pelser et al. 2007, 2010; Tang et al. 2014; Tong et al. 2017; Li et al. 2018; Zhao et al. 2023; Jin et al. 2024). The newly-described species *S.lhozhagensis* was regarded as a strongly supported monophyletic lineage (MLBS = 100, PP = 1.00) with its congener *S.acuminata*, confirming its taxonomic distinctiveness (MLBS = 96, PP = 0.99).

### ﻿Taxonomic treatment

#### ﻿ Synotis lhozhagensis

Taxon classificationPlantaeAsteralesAsteraceae

M.Tang sp. nov.

55AE2E35-5A8C-5C5A-BD58-0B4BE671B1FA

urn:lsid:ipni.org:names:77364872-1

Figs 1 , 2 , 3

##### Chinese name.

“luò zā hé ěr jú” (洛扎合耳菊)

##### Type.

**Figure 2.**
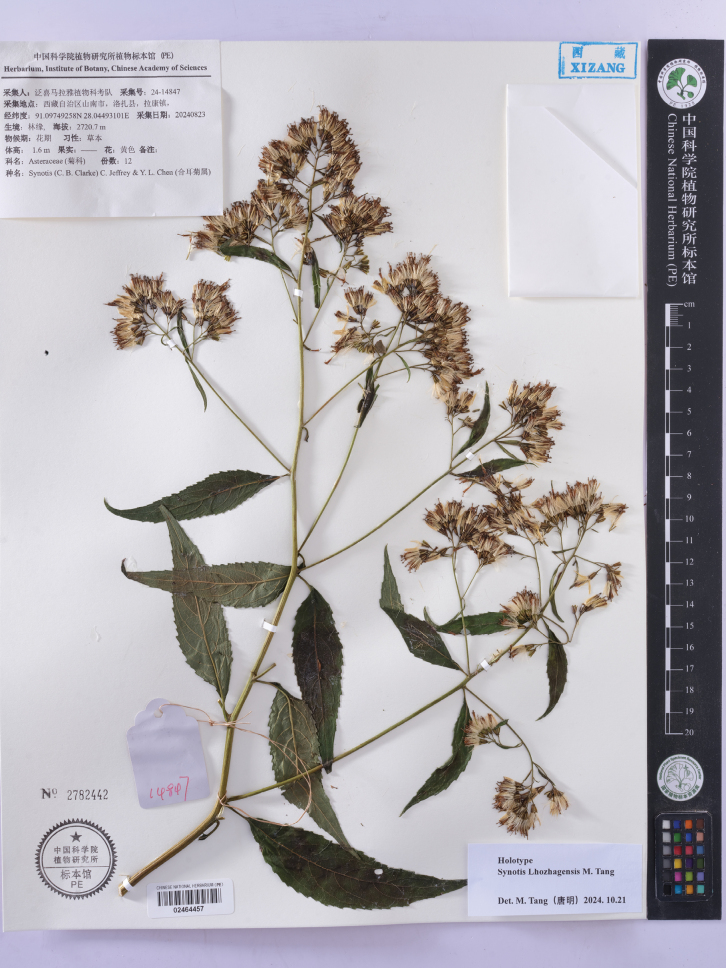
Holotype sheet of *Synotislhozhagensis*. Lakang, Lhozhag, Shannan, Xizang, China, *FLPH Expedition 24-14847* (PE).

China • Xizang Autonomous Region, Shannan City, Lhozhag County, Lakang Town, in fertile loamy soils at the edge of forests, 28°02'41.75"N, 91°05'50.97"E, 2720.7 m elev., 23 August 2024, FLPH Expedition 24-14847 (holotype, PE!; isotype, JXAU! PE!). Fig. 2.

**Figure 3.**
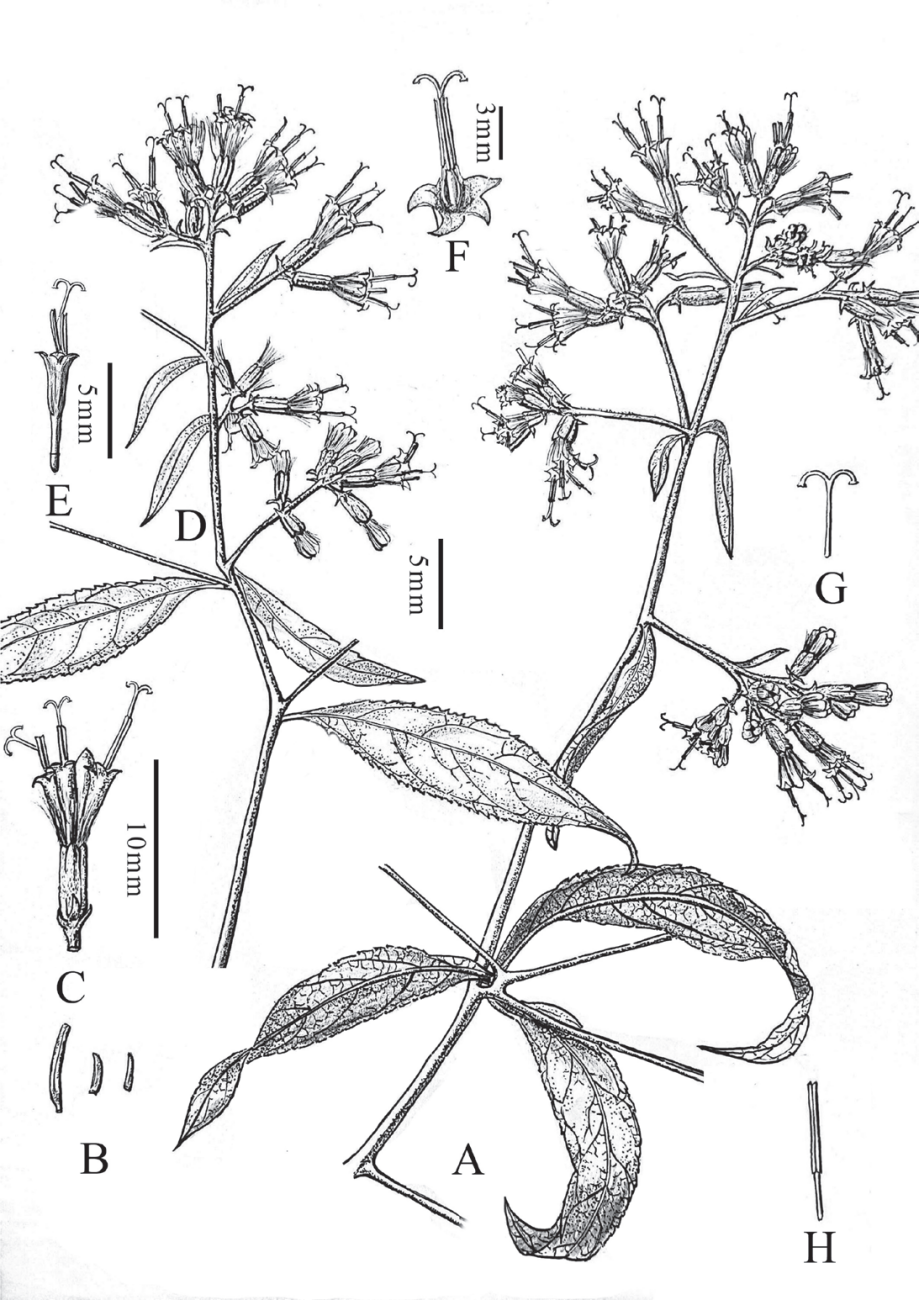
Line drawing of *Synotislhozhagensis*. **A.** Habit; **B.** Outer phyllaries (bracts of calyculus); **C.** Capitula; **D.** Synflorescence; **E.** Disc floret; **F.** Corolla; **G.** Style; **H.** Stamen. Illustration by Guo-Xing Peng based on living field-collected material (*FLPH Expedition 24-14847*).

##### Diagnosis.

*Synotislhozhagensis* is similar to *S.acuminata*, but differs in having phyllaries number five (vs. three or four), ray floret absent (vs. one) and disc florets five (vs. two or three).

##### Description.

Perennial herbs. Stem solitary, erect, 100–250 cm tall, slightly curved distally, profusely branched into short inflorescence branches, white-tomentose when young, becoming glabrous with age. Leaves papery, long-elliptic to oblanceolate-elliptic, 6–13 cm long, 1.5–3 cm wide; apex long-acuminate; base cuneate to attenuate; margins irregularly deeply obtuse-acuminate-serrate; adaxial surface sparsely adnate white-tomentose to nearly glabrous; abaxial surface sparsely white-tomentose; venation pinnate with 4–6 pairs of lateral veins, arcuately ascending; petiole 5–15 mm long, sparsely white-tomentose to nearly glabrous. Capitula lacking ray florets, aggregated into cymose panicles; peduncles short, 3–6 mm long, silvery-white-tomentose, bearing 1–2 basal bracts and bracteoles; bracts and bracteoles linear. Involucre terete, 4–5 mm long, 1.5–2 mm wide; bracts of calyculus 4–5, linear-subulate, up to 1/3 the length of involucre; involucral phyllaries five, oblong, 1.5 mm wide, apex acute, subleathery, margins broadly scarious, abaxially sparsely silvery-white pubescent. Disc florets five; corolla pale yellow, ca. 6–8 mm long; tube 2.5 mm long; limb funnel-form; Style branches 1–1.2 mm long, apically obtuse, covered with short papillate hairs, central hairs inconspicuous. Achenes cylindrical, 2.5 mm long, glabrous. Crown hairs 5 mm long, white.

##### Phenology.

Flowering July to August; fruiting September to October.

##### Etymology.

The specific epithet ‘*lhozhagensis*’ is derived from the type locality, Lakang Town, Lhozhag County, Shannan City, southeast Xizang Autonomous Region, China.

##### Distribution and habitat.

*Synotislhozhagensis* is currently known only from Lakang Town, Lhozhag County, Shannan City, Xizang Autonomous Region, China. This species inhabits forest edges and sparse understorey areas, primarily within mixed forests at elevations between 2,500 and 3,000 m (Fig. 5).

##### IUCN Red List Category.

*Synotislhozhagensis* is currently known only from its type locality near Lakang Town, Lhozhag County, Xizang Autonomous Region, China. Several small populations (with a total of no more than 100 plants) occur on forested hillsides within this area, thriving in well-preserved habitats with minimal anthropogenic disturbance. Following the IUCN Red List Categories and Criteria (IUCN 2022), we propose a provisional assessment of Data Deficient (DD) for this species, as it is newly described and additional populations may remain undiscovered.

##### Additional specimens of *Synotislhozhagensis* examined.

Xiongqu River Valley, La Jiao Village, Lhozhag County, Xizang, 2751 m elev., 28°03'07.30"N, 91°05'25.88"E, 17 August 2013, *Y.S. Chen et al. 13-1370* (PE01992899!, PE01992904!, PE01992905!).

##### Note.

**Figure 4.**
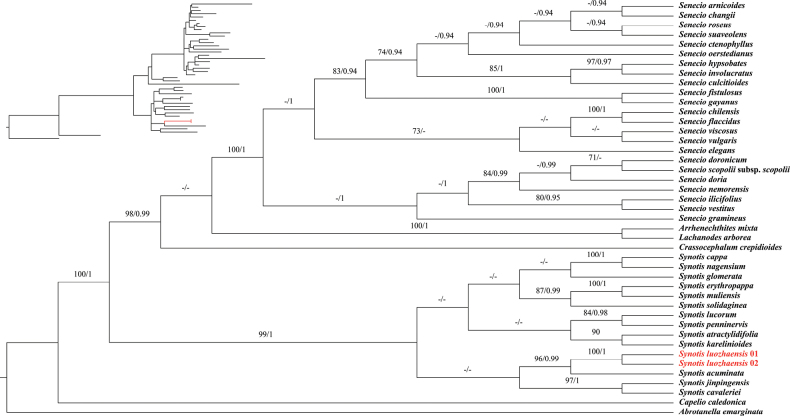
Maximum Likelihood tree for the Senecioneae based on the ITS dataset, with *Synotislhozhagensis* highlighted in red font. Bootstrap values (MLBS) and posterior probabilities (PP) are indicated above the branches. Dashes (–) indicate MLBS < 70 or PP < 0.90.

It is noteworthy that, due to the insufficient number of samples within *Synotis*, the systematic position of *Synotislhozhagensis* within *Synotis* has not been well resolved in the phylogenetic tree (Fig. 4). However, since the new species has loose corymbs that bear no ray florets, it can be easily placed under ser. Oliganthae (J.F.Jeffrey) C.Jeffrey & Y.L.Chen according to Chen et al. (2011) and Tang (2014).

**Figure 5.**
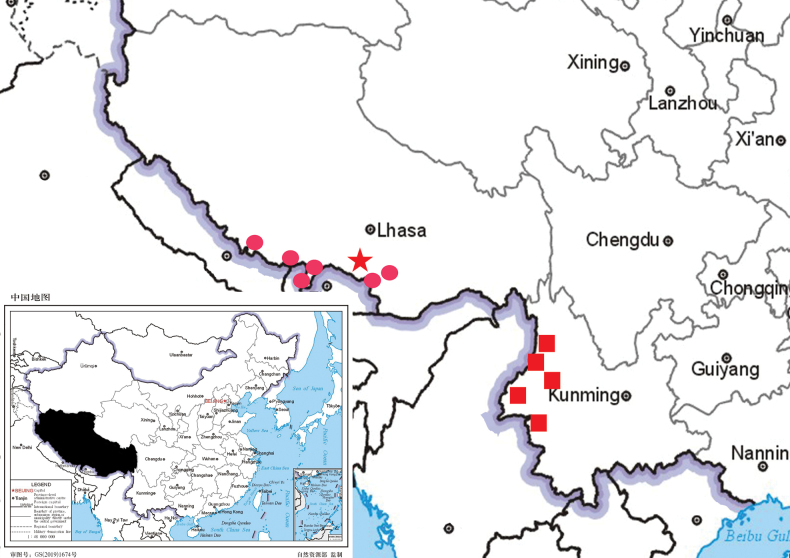
Distribution map of *Synotislhozhagensis* (red pentacle), *Synotisacuminata* (red round) and *Synotisglomerata* (red square block).

**Figure 6.**
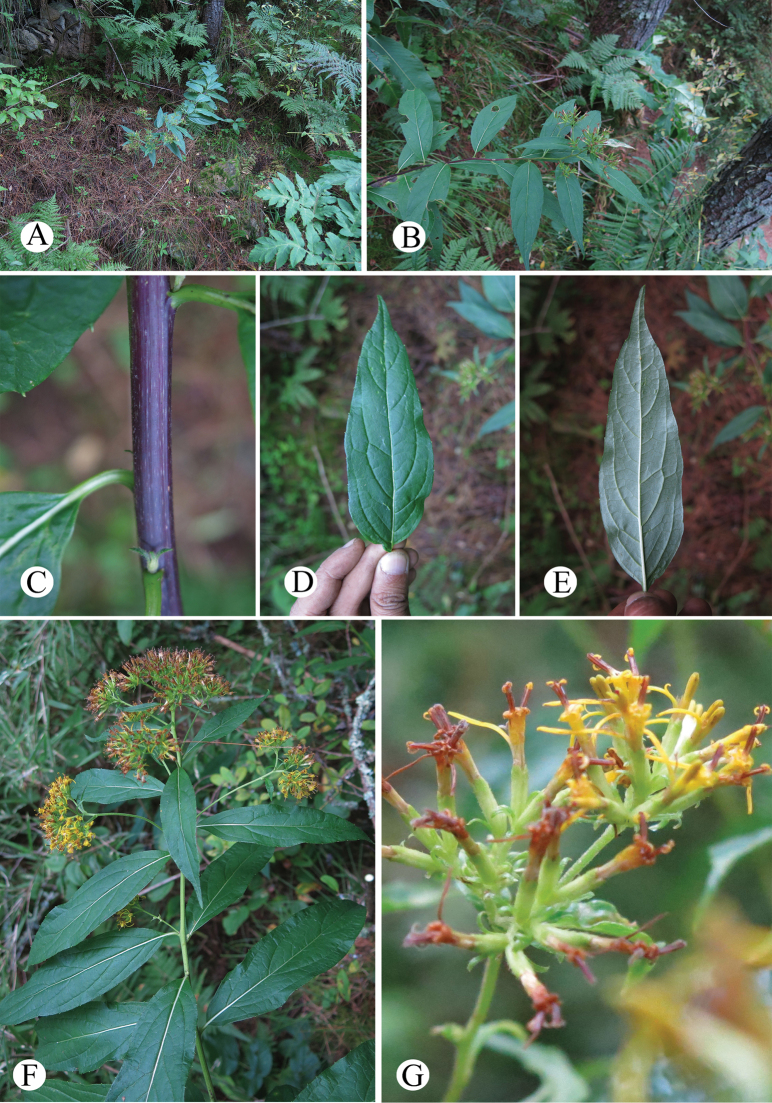
*Synotisacuminata* in the wild (Shannan, Xizang, China) **A.** Habitat; **B.** Habit; **C.** Portion of stem (sparsely tomentose); **D.** Leaf blade adaxial surface; **E.** Leaf blade abaxial surface; **F.** Synflorescence; **G.** Flowers (top view). Photographed by M. Tang.

**Figure 7.**
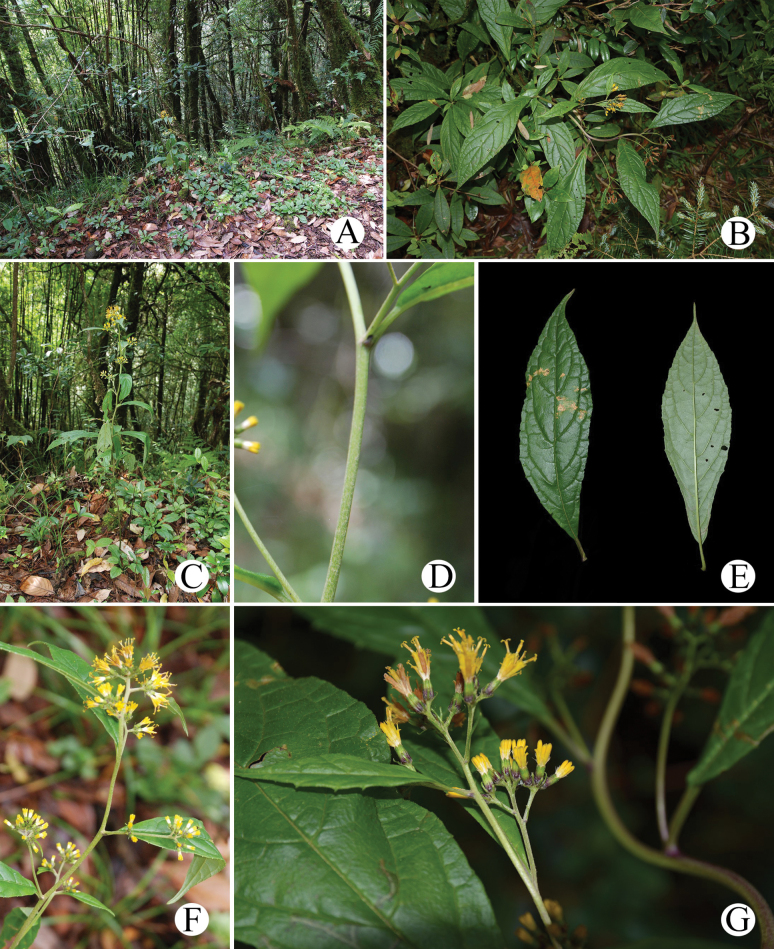
*Synotisglomerata* in the wild (Yuxi, Yunnan, China) **A.** Habitat; **B.** Habit; **C.** Habit; **D.** Portion of stem (sparsely tomentose); **E.** Adaxial (left) and abaxial (right) sides of leaf blade; **F.** Synflorescence; **G.** Flowers (top view). All photographed by M. Tang, except B, G by Y. Yang.

During our specimen review, we characterised a novel population of particular interest, i.e. *S. Noshiro et al. 9830074* (BM000810806!, E00226303!, TI!) collected in Sankhuwasabha District, Koshi Zone, Nepal on 20 August 1998, which closely matches *Synotislhozhagensis* in key features, such as plant form, leaf shape and flower clusters. However, they differ by having only two or three phyllaries. Further research is needed to confirm whether these specimens are the same as *Synotislhozhagensis* or represent another new taxon never described.

## Supplementary Material

XML Treatment for Synotis lhozhagensis

## ﻿Additional information

### Author ORCIDs

Guo-Xing Peng https://orcid.org/0009-0003-9109-0282

Liao-Cheng Zhao https://orcid.org/0000-0002-6300-7301

Ming Tang https://orcid.org/0000-0001-8419-0996

Jun Deng https://orcid.org/0009-0002-5539-0408

Hao-Ming Zhan https://orcid.org/0009-0008-2996-6040

Zhuo-Ni Zhang https://orcid.org/0009-0004-6584-0673

Qiang Wang https://orcid.org/0000-0002-0699-2034

### Data availability

All of the data that support the findings of this study are available in the main text.

## ﻿Appendix 1

**Table A1.** Voucher information and GenBank accession of species used in our study.

GenBank No. (ITS)	Taxon	Voucher Information
–	*Synotislhozhagensis* 01	*FLPH Expedition 24-14847* (PE)
–	*Synotislhozhagensis* 02	*FLPH Expedition 24-14847* (PE)
–	*Synotisacuminata*	*M. Tang591* (IBSC)
–	*Synotisglomerata*	*M. Tang181* (IBSC)
–	*Synotisjinpingensis*	*Y. Yu et al. JXAU0*1 (JXAU)
–	*Synotiscavaleriei*	*L.Y. Wang 72* (IBSC)
AF459922	*Synotisnagensium*	*B. Bartholomew et al. 1991* (-)
AY723255	*Synotislucorum*	*J.Q. Liu 2177* (HNWP)
EF538402	*Synotiscappa*	*Tessier-Yandell 86* (L)
KU696133	*Synotiserythropappa*	*C. Ren 150* (IBSC)
KU696135	*Synotismuliensis*	*C. Ren 148* (BSC)
KY347902	*Synotispenninervis*	*M. Tang & C. Ren 626* (BSC)
MH371137	*Synotisatractylidifolia*	*M. Tang & L. Wang 361* (IBSC)
MH371138	*Synotiskarelinioides*	*H.M. Li& O.G. Mao 325* (IBSC)
KX549950	*Synotissolidaginea*	*2015XZ001-B* (IBSC)
EF538298	*Senecioarnicoide*	O. *Zollner3474* (L)
AF459925	*Senecioviscosus*	*P.B. Pelser 300* (TEX)
AF459937	*Senecionemorensis*	*P.B. Pelser cult.102* (L)
AF459946	*Seneciodoria*	*P.B. Pelser cult. 129* (WIS)
EF538222	*Seneciosuaveolens*	*Elliott D.C. Dister s.n.* (MJG)
EF538298	*Senecioarnicoide*	*O. Zollner 3474* (L)
EF538312	*Senecioculcitioides*	*B. Ollgaard & H. Balslev 8822* (U)
EF538313	*Seneciochilensis*	*O. Zöllner 2958* (L)
EF538322	*Senecioctenophyllus*	*Phil. O. Zoellner 3959* (U)
EF538348	*Seneciohypsobates*	*Wedd B. Ollgaard & H. Balslev 9863* (U)
EF538362	*Seneciooerstedianus*	*B. Nordenstam 9160* (S)
EF538373	*Senecioroseus*	*J. Garcia P. 250* (L)
GU818642	*Senecioelegans*	*Cron & Goodman 687* (-)
GU818649	*Seneciogayanus*	*M. Rosas 2157* (-)
GU818650	*Seneciogramineus*	*F.K. Hoener 2104* (-)
EF538150	*Senecioinvolucratus*	*B. Nordenstam 9438* (L)
GU818650	*Seneciogramineus*	*Harv. F.K. Hoener 2104* (-)
GU818662	*Senecioilicifolius*	*Cron & Goodman 686* (-)
GU818708	*Seneciovestitus*	*P. J. Bergius W. Greuter 21766* (-)
JX895384	Senecioscopolisubsp.scopolii	–
KU499905	*Seneciochangii*	*C. Ren et al. WL146* (IBSC)
KX280815	*Seneciodoronicum*	*F. Schuhwerk 97/363* (M)
EF538143	*Abrotanellaemarginata*	*R.N.P. Goodall & J. Wood 3352* (MU)
EF538156	*Arrhenechthitesmixta*	*M.E. Lawrence 1308* (S)
EF538173	*Crassocephalumcrepidioides*	*B. Nordenstam &R. Lundin 562* (S)
GU818508	*Capeliocaledonica*	*B. Nordenstam 9644* (S)
GU818574	*Lachanodesarborea*	*R. Cairns-Wicks s.n.* (-)
